# Chemical Characteristics and Cytotoxicity to GC-2spd(ts) Cells of PM_2.5_ in Nanjing Jiangbei New Area from 2015 to 2019

**DOI:** 10.3390/toxics11020092

**Published:** 2023-01-18

**Authors:** Pengxiang Ge, Zhengjiang Liu, Mindong Chen, Yan Cui, Maoyu Cao, Xiaoming Liu

**Affiliations:** 1 Jiangsu Key Laboratory of Atmospheric Environment Monitoring and Pollution Control, Collaborative Innovation Center of Atmospheric Environment and Equipment Technology, School of Environmental Science and Engineering, Nanjing University of Information Science & Technology, Nanjing 210044, China; 2 Gansu Water Resources and Hydropower Survey and Design Research Institute, Lanzhou 730000, China

**Keywords:** PM_2.5_, cell viability, reactive oxygen species, DNA damage, source contribution

## Abstract

PM_2.5_ is an air pollutant with complex components. After entering the body through respiration, PM_2.5_ can not only cause respiratory diseases, but also break through the blood–testis barrier and influence the reproductive system. PM_2.5_ with different components may result in different toxic effects. In the first five years of Nanjing Jiangbei New Area, industrial transformation would change the concentration and chemical fraction of PM_2.5_ in the local environment to a certain extent. In this study, PM_2.5_ collected in Nanjing Jiangbei New Area every autumn and winter from 2015 to 2019 was analyzed. PM_2.5_ concentration generally decreased year by year. The large proportion of secondary inorganic ions indicated the presence of secondary pollution at the sampling site. PM_2.5_ was mainly emitted from fossil fuel combustion and vehicle exhaust. The cytotoxicity of PM_2.5_ samples was evaluated by PM_2.5_ exposure to mouse spermatocytes (GC-2spd(ts) cells). Cell viability was relatively low in 2016 and 2018, and relatively high in 2017 and 2019. Reactive oxygen species levels and DNA damage levels followed similar trends, with an overall annual decrease. The cytotoxicity of PM_2.5_ on GC-2spd(ts) cells was significantly correlated with water-soluble ions, water-soluble organic carbon, heavy metals and polycyclic aromatic hydrocarbons (*p* < 0.01). According to principal component analysis and multiple linear regression, fossil fuel combustion, secondary transformation of pollutants and construction dust were identified as the major contributors to cytotoxic effects, contributing more than 50%.

## 1. Introduction

Rapid modernization and energy consumption have led to increased pollutant emissions, and air pollution is gradually becoming a critical environmental problem in China [1]. Air pollution can adversely affect human health and increase the risk of cancer [2]. PM_2.5_, an important air pollutant, was recognized as a class I carcinogen by the International Agency for Research on Cancer (IARC), and its concentration is an important indicator of air pollution [3,4]. PM_2.5_ pollution has become a major global environmental problem [5]. PM_2.5_ was ranked as the fifth leading cause of death in the 2015 Global Burden of Disease Study, with PM_2.5_ exposure resulting in more than 4 million premature deaths [6]. According to National Class I standard of PM_2.5_ concentration of 35 μg/m^3^, 81% of Chinese residents lived in areas where PM_2.5_ concentration exceeded the standard in 2017 [7]. A growing number of toxicological studies have demonstrated that PM_2.5_ can enter and deposit in the alveoli carrying numerous chemicals, bacteria and viruses [8]. PM_2.5_ can directly affect the respiratory system by inducing oxidative damage and inflammatory responses, and can also reach other organs through blood circulation, leading to serious multisystem diseases [9]. PM_2.5_ exposure can cause vascular endothelial cell damage, lead to cellular dysfunction and increase the risk of cardiovascular diseases such as atherosclerosis [10]. A large number of toxicological studies related to PM_2.5_ have focused on the respiratory and cardiovascular systems [11]. However, PM_2.5_ with smaller particle size can break through the blood–testis barrier and blood–brain barrier after entering the human body, thereby influencing the reproductive system and the nervous system [12]. In recent years, reproductive toxicity research has gradually attracted attention and is considered an important part of PM_2.5_ toxicity research. It has been indicated that PM_2.5_ can inhibit cell viability (CV) by increasing the level of reactive oxygen species (ROS) in mouse spermatocytes (GC-2spd(ts) cells), thereby reducing the quantity and quality of the sperm [13]. In addition, PM_2.5_ exposure can lead to DNA damage in GC-2spd(ts) cells and induce apoptosis [14]. However, studies on the cytotoxicity of PM_2.5_ are still rare, and most of the studies on GC-2spd(ts) cells have focused on the toxicity of nanomaterials such as nano-silica [15,16]. In vitro exposure studies of PM_2.5_ on GC-2spd(ts) cells can reflect the reproductive toxicity of PM_2.5_ to a certain extent, and they make it easier to explain the toxicogenic mechanisms involved.

PM_2.5_ in different regions and time leads to different disease burden, and the toxicity of PM_2.5_ with various chemical components varies [17]. Water-soluble substances are the main chemical components in PM_2.5_. Water-soluble ions (WSI) and water-soluble organic carbon (WSOC) can affect cell viability and ROS generation [18,19,20,21]. Organic compounds such as polycyclic aromatic hydrocarbons (PAHs) can induce cellular oxidative stress, DNA damage and inflammatory responses [22,23]. Heavy metals (HMs) in PM_2.5_ can promote intracellular oxidative stress, destroy cell structure and decrease cell viability [24,25]. Heavy metals are potentially reproductive toxic as they can accumulate in organisms and affect the development and normal function of the reproductive system [26]. Due to the change of economic and industrial structure and the intervention of a series of control measures, the characteristics of atmospheric particulate matter in China were gradually changing. From 2005 to 2012, sulfate concentrations in PM_2.5_ in China decreased, while nitrate concentrations continued to increase [27]. From 2013 to 2017, PM_2.5_ concentrations decreased by 28–40% in different regions of China, and SO_2_, NO_x_ and OC emissions nationwide declined by 59%, 21% and 32%, respectively [28]. It has been reported from a study in the Beijing-Tianjin-Hebei region that interannual variation in regional meteorological conditions is one of the reasons for the changes in PM_2.5_ concentrations and fractions [29]. In the first five years of Nanjing Jiangbei New Area (2015–2019), the economic structure changed, with fewer traditional chemical industries and more high-tech industries. During this transition process, the concentration and composition of PM_2.5_ were supposed to change, and the health risk to approximately two million residents in the new area may vary as well. The chemical fraction and potential toxicity of PM_2.5_ in these five years can reflect the impact of the construction of the new area on the surrounding environment and the health of residents. It is also conducive to the formulation and implementation of subsequent environmental policies. In this study, the chemical fractions of PM_2.5_ collected every autumn and winter from 2015 to 2019 in Nanjing Jiangbei New Area were determined. Moreover, the reproductive toxicity of PM_2.5_ samples was evaluated by analyzing the exposure toxicity of PM_2.5_ samples to mouse spermatocytes in vitro.

## 2. Materials and Methods

### 2.1. Sampling and Pretreatment of PM_2.5_

PM_2.5_ samples were collected on the roof of the library of Nanjing University of Information Science and Technology, Jiangbei New Area, Nanjing, China (Figure S1). From 2015 to 2019, 20 samplings were carried out from October to November each year, with a total of 100 samples. PM_2.5_ was collected on quartz microfiber filters (Whatman, UK) through a large-flow sampler (Tisch Environmental, Cleves, OH, USA) with a flow rate of 1.13 m^3^/min. The filters were preheated in a muffle furnace at 450 °C for 6 h to remove impurities. Before and after sampling, the filters were weighed after standing in a desiccator at room temperature for 24 h. The sampled filters were cut into pieces according to different years and extracted ultrasonically with ultrapure water for 20 min three times. The suspension was filtered through eight layers of gauze and freeze-dried to obtain PM_2.5_ powder. The samples were stored in a refrigerator at −20 °C away from light for later use.

### 2.2. Composition Analysis of PM_2.5_

After microwave digestion with 65% HNO_3_, the concentrations of heavy metals (including Pb, Zn, Ba, Cu, Sr, Mn, Cr, Ni and Cd) in the samples were determined by an inductively coupled plasma mass spectrometry (ICP-MS, Thermo Fisher Scientific, Waltham, MA, USA). After sonication with ultrapure water and filtration through the polytetrafluoroethylene (PTFE) membranes with a pore size of 0.22 μm, the concentrations of the water-soluble ions (including Na^+^, NH_4_^+^, K^+^, Mg^2+^, Ca^2+^, F^−^, Cl^−^, SO_4_^2−^ and NO_3_^−^) and WSOC in the samples were determined by an ion chromatograph (IC, Dionex, Sunnyvale, CA, USA) and a total organic carbon analyzer (TOC-L, Shimadzu, Tokyo, Japan), respectively. After adding the internal standard solution and dichloromethane, the samples were subjected to ultrasonic filtration and rotary evaporation. Then, PAHs concentrations in the samples were measured by a gas chromatography-mass spectrometer (GC-MS, Agilent, Santa Clara, CA, USA). According to the list of class I and class II carcinogens published by IARC, 15 PAHs were detected in this study, including naphthalene (Nap), acenaphthylene (AcPy), acenaphthene (Acp), fluorene (Flu), phenanthrene (PA), anthracene (Ant), fluoranthene (FL), pyrene (Pyr), benzo[a]anthracene (B[a]A), chrysene (CHR), benzo[b&k]fluoranthene (B[b&k]F), benzo[a]pyrene (B[a]P), indeno[1,2,3-cd]pyrene (IND), Dibenzo[a,h]anthracene (DBA) and Benzo[g,h,i]perylene (B[ghi]P). The standard curves of all tested substances were linear (r^2^ > 0.997). The blank samples with standard reference were analyzed in parallel with tested samples. The recovery rates of all tested substances were within 100 ± 15%. Further method details and instrumental conditions were given in Tables S1–S3.

### 2.3. Cell Culture and Exposure

GC-2spd(ts) cells were used in this study, which were kindly provided by Stem Cell Bank, Chinese Academy of Sciences. GC-2spd(ts) cells were cultured in Dulbecco’s Modified Eagle Medium (DMEM, Bio-Channel, China) supplemented with 10% fetal bovine serum (FBS, Gibco, Australia), and maintained in an incubator (Thermo Scientific, Waltham, MA, USA) at a constant temperature of 37 °C and 5% CO_2_. The PM_2.5_ powder was dissolved with serum-free DMEM to prepare the PM_2.5_ venom with a certain concentration gradient (0, 50, 100, 200, 400 µg/mL) after ultrasonication and vortexing. The concentrations of the PM_2.5_ venom were selected based on the results of previous experiments. At this concentration gradient, the cells exhibited variable toxic effects while ensuring a certain viability. For each exposure experiment, GC-2spd(ts) cells in logarithmic growth stages were seeded at a density of 1 × 10^5^ cells/mL and cultured for 24 h, and then exposed to PM_2.5_ at different concentrations for 24 h. A blank group and a control group were set up in each experiment. The blank group contained only DMEM without cells and PM_2.5_, while the control group contained only DMEM and cells without PM_2.5_. Each sample was repeated three times in parallel.

### 2.4. Cytotoxicity Assay

In this study, cell viability, cellular ROS levels and DNA damage levels of GC-2spd(ts) cells were measured after exposure to PM_2.5_. Cell viability was detected using the CCK-8 kit (Beyotime Biotechnology, Shanghai, China). First, 10 μL of CCK-8 reagent was added to each sample. After incubation at 37 °C in the dark for 2 h, the optical density (OD) of the samples was measured at the wavelength of 450 nm by a microplate reader (Molecular Devices, San Jose, CA, USA). Cell viability is expressed as the ratio of (OD_sample_-OD_blank_) to (OD_control_-OD_blank_). Cellular ROS levels were detected using the DCFH-DA probe. The DCFH-DA powder (Sigma-Aldrich, St. Louis, MO, USA) was dissolved in dimethyl sulfoxide (DMSO, Purity > 99.9%, Beyotime Biotechnology, Shanghai, China) to prepare a 10 mM stock solution. It was then diluted with serum-free DMEM to 10 µM DCFH-DA solution (0.1% DMSO) and added to each sample. After incubation at 37 °C in the dark for 25 min, the cells were washed three times with phosphate-buffered saline (PBS) and collected. The collected cells were measured for fluorescence intensity by flow cytometry (Beckman, Brea, CA, USA) at the excitation wavelength of 488 nm and emission wavelength of 525 nm. The cellular ROS level was expressed as the ratio of fluorescence intensity of the sample group to the control group. For the DNA damage assay, DNA double strand break staining kit by γ-H2AX immunofluorescence (Beyotime Biotechnology, Shanghai, China) was used. When cellular DNA double strand breaks, H2AX will undergo phosphorylation modification to produce γ-H2AX. Therefore, γ-H2AX is often used as a marker of DNA damage, whose content level can reflect the degree of cellular DNA damage. After staining with the kit, the cells were observed and captured under a fluorescence microscope (Jiangnan Yongxin Optics, Nanjing, China). γ-H2AX exhibited green fluorescence at the excitation wavelength of 488 nm. The fluorescence intensity was calculated by the captured images and ImageJ software (v1.52, National Institutes of Health, Bethesda, MD, USA). The DNA damage level was expressed as the ratio of the fluorescence intensity of the sample group to the control group. All experiments were performed in three replicates to ensure the accuracy of the results.

### 2.5. Principal Component Analysis-Multiple Linear Regression (PCA-MLR)

Principal component analysis (PCA) was performed on the chemical components of PM_2.5_ in Nanjing Jiangbei New Area using SPSS software (v23.0, International Business Machines Corporation, Armonk, NY, USA). Several principal components that can represent the vast majority of the variation in the samples would be extracted. The sources of PM_2.5_ were identified by using the principal factor loadings of the chemical elements after great rotation of the variance. Multiple linear regression analysis (MLR) was then performed by SPSS software (v23.0) to obtain the main sources and their relative contributions.

### 2.6. Statistical Analysis

Statistical analysis was performed using SPSS software (v23.0). The variation of cytotoxicity at different exposure concentrations was analyzed by one-way analysis of variance (ANOVA). The correlation between the composition and cytotoxicity of PM_2.5_ was investigated by Pearson correlation analysis, and the results are shown in Figure S2. For all cases, *p* < 0.05 was considered statistically significant.

## 3. Results and Discussion

### 3.1. Chemical Characteristics of PM_2.5_

**Figure 1:**
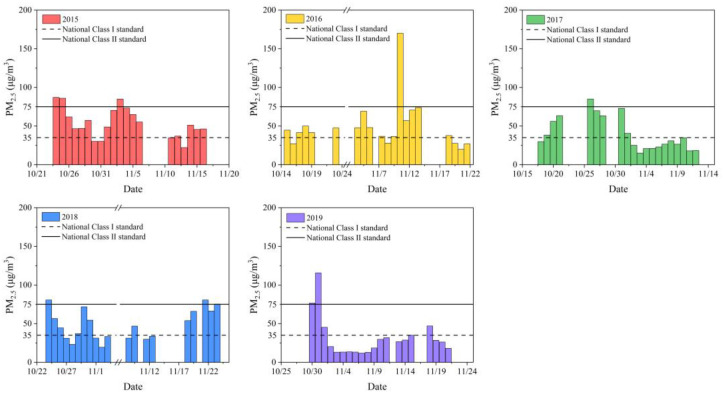
Daily PM_2.5_ concentrations in Nanjing Jiangbei New Area during the sampling period from 2015 to 2019.

The average PM_2.5_ concentration of Nanjing Jiangbei New Area during the sampling period from 2015 to 2019 was 54.00 ± 18.68 µg/m^3^, 50.08 ± 31.19 µg/m^3^, 38.97 ± 20.87 µg/m^3^, 48.35 ± 19.25 µg/m^3^ and 31.28 ± 24.68 µg/m^3^, respectively. As shown in Figure 1, during the sampling period from 2015 to 2019, the number of days that the daily average PM_2.5_ concentration reached the national class I standard was 4, 5, 12, 8 and 16 days, respectively. Overall, PM_2.5_ concentrations during the sampling period showed a decreasing trend year by year. This may be related to the industrial transformation of Nanjing Jiangbei New Area after its establishment in 2015, where high-tech industries have gradually replaced the traditional chemical industry. In addition, the rebound of PM_2.5_ concentrations in 2018 may be due to extreme meteorological conditions and temperature inversion, which were unfavorable to the diffusion of pollutants. After the sampling period of this study in 2018, a heavy haze event lasting for two weeks occurred in Nanjing. As a result, the air pollution control for the autumn and winter seasons of 2019 was advanced to the beginning of October, which contributed to the lowest PM_2.5_ concentration during the sampling period in 2019. Since the composition of PM_2.5_ varies with the emission sources, seasons, and climate, PM_2.5_ concentrations do not adequately explain the risk to human health. Therefore, the contents of water-soluble components, heavy metals and PAHs in sampled PM_2.5_ were determined in this study. The mass proportions of major chemical compositions in PM_2.5_ are shown in Figure S3. Water-soluble substances were the measured components with the highest proportion of PM_2.5_, accounting for 45.54–64.26%, which was similar to the previous result [30]. Considered with PM_2.5_ concentrations, all measured components had the greatest abundance in the atmosphere in 2015. The unidentified fraction is estimated to be the insoluble carbonaceous component.

#### 3.1.1. Water-Soluble Ions

**Figure 2:**
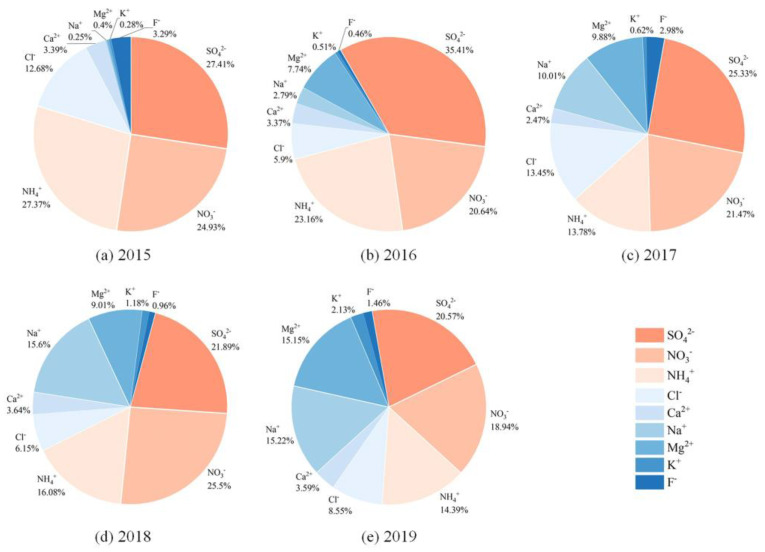
The relative proportion of each water-soluble ion in the total measured water-soluble ions. SO_4_^2−^, NO_3_^−^ and NH_4_^+^ were the most abundant water-soluble ions in PM_2.5_ in Nanjing Jiangbei New Area from 2015 to 2019.

The proportion of water-soluble ions in PM_2.5_ samples from 2015 to 2019 ranged from 31.63% to 52.94%, which was higher than that in Xiamen (24.4%) and lower than that in Zhengzhou (66.1%) [31,32]. As shown in Figure 2, SO_4_^2−^, NO_3_^−^ and NH_4_^+^ (SNA) were the most abundant water-soluble ions, accounting for 53.89–79.71% of the total water-soluble ions. SNA can affect atmospheric visibility and acidity of precipitation, adversely influencing regional climate and the lives of residents [33,34]. SNA are mainly generated through the secondary transformation of SO_2_, NO_x_ and NH_3_. The high SNA concentrations in PM_2.5_ samples from 2015 to 2019 indicated relatively severe secondary pollution around the sampling sites. The ratio of NO_3_^-^ concentration to SO_4_^2-^ concentration is often used to identify whether NO_x_ and SO_2_ in the atmosphere come from mobile or fixed sources [35]. If the ratio is greater than 1, the emission sources are dominated by mobile sources (e.g., vehicle exhaust) and conversely by fixed sources (e.g., coal combustion). The ratios of PM_2.5_ samples from 2015 to 2019 were 0.91, 0.58, 0.85, 1.16 and 0.92, respectively. Except for that in 2018, the NO_3_^−^/SO_4_^2−^ in other years were less than 1, indicating that NO_x_ and SO_2_ in Nanjing Jiangbei New Area in autumn and winter were mainly from fixed source emissions. However, the values of NO_3_^−^/SO_4_^2−^ generally showed an increasing trend, suggesting that the contribution of mobile source emissions to PM_2.5_ pollution was increasing. NH_4_^+^ was significantly correlated with SO_4_^2−^ and NO_3_^−^, with correlation coefficients of 0.91 and 0.95, respectively (*p* < 0.01). The ratios of the molar concentration of NH_4_^+^ to SO_4_^2−^ in sampled PM_2.5_ for five years were all calculated to be greater than 2, determining that NH_4_^+^ was present in the atmosphere primarily in the form of ammonium sulfate and ammonium nitrate [36]. Na^+^ and Cl^−^ were also present in relatively high levels, which are generally considered to come from the ocean. However, the correlation between Na^+^ and Cl^−^ in this study was not significant, implying that there may be other sources. The concentration ratio of Cl^−^ to Na^+^ in seawater is 1.8 [37], which in sampled PM_2.5_ from 2015 to 2019 were 50.95, 2.11, 1.34, 0.39 and 0.56, respectively. Therefore, it was presumed that Cl^−^ in PM_2.5_ in 2015 and 2016 were likely from fossil fuel combustion [38], while Na^+^ were from biomass combustion [39]. In contrast, Na^+^ and Cl^-^ in PM_2.5_ mainly came from the ocean in 2017, 2018 and 2019. In addition, F^-^ was emitted from fossil fuel combustion [40], K^+^ was mainly from biomass combustion [41], Ca^2+^ was mostly influenced by construction dust [42] and both the latter two sources contributed to Mg^2+^ [43].

#### 3.1.2. Heavy Metals

**Figure 3:**
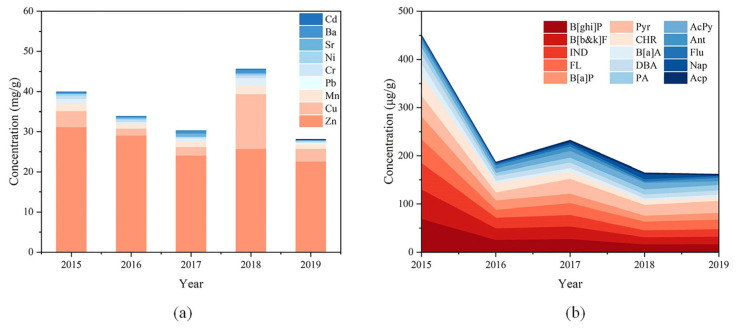
(**a**) Mass concentrations of heavy metal elements in PM_2.5_ from 2015 to 2019. The highest content of heavy metals in sampled PM_2.5_ was found in 2018, while the content of heavy metals decreased year by year in other years. (**b**) Mass concentrations of PAHs in PM_2.5_ from 2015 to 2019. The total PAHs concentration in general tended to decrease.

The fractions of heavy metals in sampled PM_2.5_ from 2015 to 2019 were 3.99%, 3.38%, 3.02%, 4.56% and 2.81%, respectively. The highest content of heavy metals in sampled PM_2.5_ was found in 2018, while the content of heavy metals decreased year by year in other years. The concentration of each metal element varied somewhat from year to year, which may be related to its source. Zn and Cu are mainly from metallurgical industry and fuel combustion [44,45]. Vehicle exhaust and parts wear and are also important sources of Zn and Cu [46,47]. Mn and Ba have high background values in soil and generally come from ground dust [48,49,50]. Pb, Cr and Ni are usually emitted through fossil fuel combustion and vehicle exhaust [51]. Sr may come from incineration soot and ground dust [52], while Cd is largely from combustion of coal and fuel oil [53]. In these five years, Zn, Cu, Mn and Pb were the most abundant metal elements in the sampled PM_2.5_, accounting for 93.43–97.05% of the total measured heavy metals (Figure 3a), which was consistent with the result of previous research [54]. This may be related to the reserved chemical parks, the new construction sites, and the large number of transportation vehicles during the construction phase of Nanjing Jiangbei New Area. To further distinguish the sources of metal elements, enrichment factor analysis was used to determine the contribution of natural and anthropogenic sources to the metal elements in sampled PM_2.5_ [55]. In this study, Mn was selected as the reference element to calculate the enrichment factors (EF) of the other eight metal elements, and the results were listed in Table S4. Zn, Cu and Cd were dominated by anthropogenic sources. Ba was mainly from natural sources. Pb, Cr and Ni were contributed by both natural and anthropogenic sources. Sr in 2016 and 2017 was contributed by a combination of natural and anthropogenic sources, while Sr in the remaining years was from natural sources. This indicated that the heavy metals in PM_2.5_ in Nanjing Jiangbei New Area from 2015 to 2019 mostly came from emissions of chemical parks and road traffic. During the sampling period from 2015 to 2019, the concentrations of Zn in PM_2.5_ showed an overall decreasing trend, while Cu was increasing, indicating that Zn in the atmosphere mainly came from chemical industry emissions, while Cu was more likely to be from vehicle exhaust and parts wear.

#### 3.1.3. Organic Carbon

Organic carbon is an important component of PM_2.5_, including primary organic carbon (POC) emitted directly by combustion and secondary organic carbon (SOC) generated by volatile organic compounds (VOCs) through photochemical reactions [56,57]. In general, organic carbon accounts for about 50% of the total mass of PM_2.5_, with WSOC occupying 10–70% of organic carbon [58,59,60]. The concentrations of WSOC in PM_2.5_ in Nanjing Jiangbei New Area during the sampling period from 2015 to 2019 ranged from 9.46% to 19.76%, with the highest and lowest concentrations appearing in 2015 and 2018, respectively. In this study, there was a highly significant correlation between WSOC and Cl^−^ (r^2^ = 0.94, *p* < 0.01), suggesting that fossil fuel combustion contributed to WSOC in PM_2.5_. Meanwhile, WSOC also exhibited relatively strong correlation with SNA, with correlation coefficients of 0.77, 0.82 and 0.83, respectively (*p* < 0.01). As mentioned above, there was serious secondary pollution at the sampling sites during the sampling period, thus WSOC may be partially generated through secondary reactions. In addition, the concentrations of PAHs in sampled PM_2.5_ were measured in this study, as shown in Figure 3b. The concentrations of PAHs in sampled PM_2.5_ from 2015 to 2019 ranged from 161.93 to 450.70 ng/g, accounting for a mere 0.02–0.05%. Despite their low levels in PM_2.5_, PAHs are carcinogenic and can influence human reproductive function as endocrine disruptors [23,61]. In this study, the total PAHs concentration in general tended to decrease. The 4–6-ring PAHs were the most abundant, accounting for 72.17–90.97% of the total PAHs, but their concentrations displayed a yearly downward trend as well. For each year, B[ghi]P was the most abundant single PAH, followed by B[b&k]F and IND. These high molecular weight PAHs are generally considered to be derived from fuel and coal combustion [62]. To further identify the sources of PAHs in PM_2.5_, the diagnostic ratio analysis was utilized, and the results were listed in Table 1 [63,64,65]. According to that, PAHs in PM_2.5_ during the sampling period from 2015 to 2019 were mainly emitted from combustion sources, including coal, diesel and gasoline. Moreover, PAHs with high concentrations were significantly correlated with Cl^−^ and Zn from industrial sources, but weakly correlated with NO_3_^-^ and Cu from traffic sources. Therefore, PAHs in PM_2.5_ were majorly contributed by fossil fuel combustion in the surrounding chemical parks in this study.

### 3.2. Cytotoxicity of PM_2.5_

#### 3.2.1. Cell Viability

**Figure 4:**
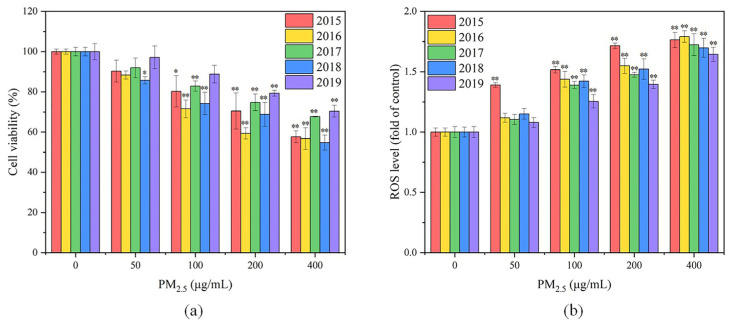
(**a**) Cell viability and (**b**) ROS levels of GC-2spd(ts) cells after PM_2.5_ exposure for 24 h. Compared with the control group, * means *p* < 0.05, ** means *p* < 0.01.

As shown in Figure 4a, the viability of GC-2spd(ts) cells in all five exposure groups decreased with increasing PM_2.5_ exposure concentrations. At the PM_2.5_ exposure concentration of 50 μg/mL, the cell viability of the exposure group was not significantly different from that of the control group. When the PM_2.5_ concentration was increased to 100–200 μg/mL, the cell survival rates of the exposure groups varied significantly from the control group. The highest cytotoxicity of PM_2.5_ was presented in 2016, followed by 2018, 2015, 2017 and 2019. At the exposure concentration of 400 μg/mL, the lowest and highest cell survivals were obtained in 2018 and 2019, which were 54.73% and 70.38%, respectively. A linear fit was performed for PM_2.5_ exposure concentration and cell viability for each year from 2015 to 2019. It was found that there was a linear negative correlation between PM_2.5_ exposure concentration and cell viability for each year (*p* < 0.05), with r^2^ of 0.93, 0.76, 0.88, 0.88 and 0.94, respectively, demonstrating a dose–effect relationship between PM_2.5_ exposure and cell viability. Similarly, a study in Beijing revealed that PM_2.5_ inhibited the viability of GC-2spd(ts) cells and even induced apoptosis at high exposure concentrations [14]. It has also been suggested that these may be due to the elevated cellular ROS level caused by PM_2.5_ [13]. Overall, the cytotoxicity of PM_2.5_ in 2017 and 2019 was relatively weak, whereas that in 2016 and 2018 was more able to inhibit the viability of GC-2spd(ts) cells, probably due to the differences in the chemical compositions of PM_2.5_ in different years. The highest SNA level in 2016 and the highest heavy metal level in 2018 may contribute to the stronger cytotoxicity of PM_2.5_ in these two years.

#### 3.2.2. Cellular ROS

As shown in Figure 4b, the ROS levels of GC-2spd(ts) cells increased with increasing exposure concentrations in the five exposure groups from 2015 to 2019. At the low exposure concentration (50 μg/mL), there was no significant difference in cellular ROS levels between the exposure group and the control group, except for the 2015 exposure group. However, when the exposure concentration was increased to 100 μg/mL, the cellular ROS levels of all exposure groups were significantly different from that of the control group (*p* < 0.05), indicating that oxidative stress occurred intracellularly in all exposure groups. At concentrations of 50–200 μg/mL, PM_2.5_ in 2015 led to the highest ROS levels, which significantly varied from the results of the exposure group in other years (*p* < 0.05). The highest WSOC concentration, the highest PAHs concentration and relatively high heavy metal concentration were found in PM_2.5_ in 2015, and all these components are believed to induce elevated cellular ROS [21,22,23,24,25]. The lowest ROS level at each concentration was observed in the 2019 exposure group, which might be due to the lowest concentrations of water-soluble ions, heavy metals and PAHs in PM_2.5_ in 2019. As the exposure concentration increased, the elevation of cellular ROS levels became slower in all exposure groups. This could result from that the cell viability decreased with increased exposure concentrations, and the cells that were apoptotic or dead due to severe oxidative stress failed to be detected. It has also been demonstrated in another study that PM_2.5_ exposure not only caused an elevated ROS level in GC-2spd(ts) cells, but also led to cellular DNA damage [14]. By adding antioxidants, DNA damage was improved and apoptosis was inhibited, suggesting that oxidative stress played a key role in the adverse effects of PM_2.5_ on GC-2spd(ts) cells.

#### 3.2.3. DNA Damage

**Figure 5:**
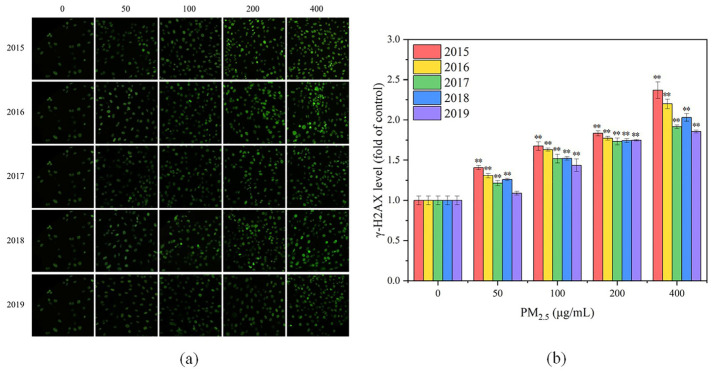
(**a**) Fluorescence images of γ-H2AX in GC-2spd(ts) cells after PM_2.5_ exposure for 24 h. From left to right, different exposure concentrations are presented for 0, 50, 100, 200 and 400 μg/mL. From top to bottom, different years for PM_2.5_ are presented for 2015, 2016, 2017, 2018 and 2019. Compared with the control group (0 μg/mL), all exposure groups exhibited more fluorescent spots and higher fluorescence intensity. (**b**) γ-H2AX levels of GC-2spd(ts) cells after PM_2.5_ exposure for 24 h. Compared with the control group, ** means *p* < 0.01.

When DNA double-strand breaks, H2AX can be phosphorylated to generate γ-H2AX [66], the level of which can reflect the degree of DNA damage. In this study, the green fluorescence of γ-H2AX could be observed under fluorescence microscope after staining, as shown in Figure 5a. Compared with the control group, all exposure groups exhibited more fluorescent spots and higher fluorescence intensity. With the increase of exposure concentration, the number and fluorescence intensity of fluorescent spots in the images increased, and the γ-H2AX content increased. This indicated that more cells underwent DNA damage and that the DNA damage was aggravated. It is evident from Figure 5a that exposure to PM_2.5_ in 2015 and 2016 induced a greater degree of DNA damage. To quantify the level of DNA damage in GC-2spd(ts) cells, the fluorescence intensity was calculated for each image, as shown in Figure 5b. With increasing PM_2.5_ concentration, the γ-H2AX level in GC-2spd(ts) cells increased significantly (*p* < 0.01), indicating an elevated level of cellular DNA damage. Zhang et al. also demonstrated that PM_2.5_ increased 8-OHdG level in GC-2spd(ts) cells at high exposure doses, inducing DNA damage, which in turn may lead to reduced fertility [13]. Overall, exposure to PM_2.5_ in 2015 resulted in the highest γ-H2AX level and the highest degree of DNA damage, followed by PM_2.5_ in 2016, and the weakest toxic effect of PM_2.5_ was observed in 2019. This trend approximated that of ROS levels, probably because intracellular oxidative stress can induce DNA damage. After DNA damage occurs in germ cells, failure to repair in time or failure to repair will lead to apoptosis or alteration of genetic information, thereby resulting in genotoxicity [67].

### 3.3. Potential Toxic Components, Sources and Contributions

There are differences in the toxic effects caused by different components of PM_2.5_. PAHs are intensely carcinogenic and can reduce cell viability, thereby threatening human health [68,69]. Heavy metals are bioaccumulative, which can influence bones and nerves, and even result in genetic damage [70,71]. PAHs and heavy metals in PM_2.5_ were confirmed by previous studies to induce DNA damage and increased cellular ROS [72,73]. Compared with other components, water-soluble components are relatively less toxic, but they can be distributed throughout the body with the blood, resulting in respiratory and cardiovascular diseases [74,75]. Water-soluble components of PM_2.5_ can lead to apoptosis by triggering inflammatory responses [76]. In this study, cytotoxicity of GC-2spd(ts) cells was significantly correlated with water-soluble ions, WSOC, heavy metals and PAHs in PM_2.5_ (*p* < 0.01). The favorable correlation suggested that the presence of these chemical components may contribute to the reduced cell viability, increased ROS levels, and enhanced DNA damage of GC-2spd(ts) cells. The differences in the chemical fraction of PM_2.5_ often depend on the source. From 2015 to 2019, the proportion of chemical components of PM_2.5_ in Nanjing Jiangbei New Area had varied, but these chemical components that probably induce toxic effects were always present. Therefore, tracing the sources of these toxicogenic components, assigning the contribution of each source to cytotoxic effects, and controlling the sources and generation pathways of toxicogenic components can reduce the health risk of PM_2.5_ to a certain extent. In this study, principal component analysis and multiple linear regression were used in combination. Five characteristic factors were extracted by PCA from various chemical components of Jiangbei New Area for five years. The contributions of the five factors to cytotoxic effects were then evaluated by MLR.

**Figure 6:**
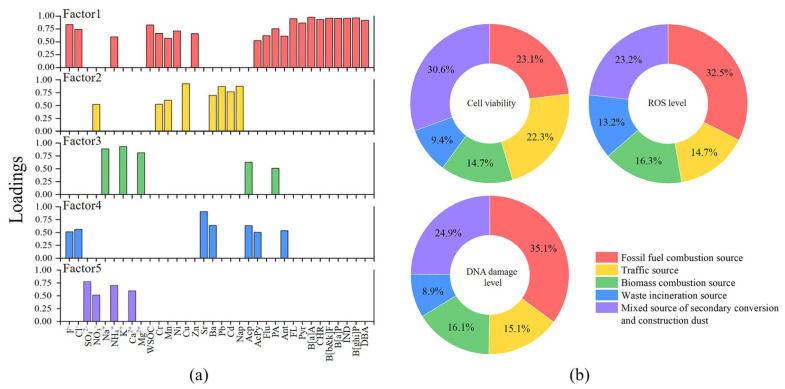
(**a**) Loadings of the main components of the five characteristic factors. (**b**) Relative contributions of the five sources to cytotoxic effects.

The cumulative variance contribution of the five characteristic factors reached 100%. The total variance explained and the rotated factor loading matrix were listed in Tables S5 and S6, respectively. The components with loadings higher than 0.5 in each factor were presented in Figure 6a. The loadings of 4–6-ring PAHs were higher in Factor 1, which were all greater than 0.8. In addition, F^−^, Cl^−^, Ni, Zn and WSOC also had large loadings in Factor 1. Combined with the previous analysis, Factor 1 mainly represented fossil fuel combustion source. In Factor 2, Cu, Pb, Cd and Nap exhibited strong loadings. Cu, Pb and Cd are considered to come from vehicle exhaust and parts wear, while Nap can be released from diesel exhaust of transport vehicles and asphalt used for paving [76]. NO_3_^−^ also had a large loading in Factor 2, and its precursor (NO_x_) was mainly from vehicle exhaust, indicating that Factor 2 primarily represented traffic source. The loadings of Na^+^, K^+^ and Mg^2+^ were larger in Factor 3, and they were considered to be mainly generated by biomass combustion in this study. Factor 3 was defined as biomass combustion source. The high loadings of Sr and Ba were found in Factor 4, which were initially considered to be from ground dust. However, F^−^, Cl^−^, Acp, AcPy and Ant also presented moderate loadings, and these components are usually common species in combustion products. Sr was possibly from the soot of waste incineration [77], and Ba can be detected in large quantities in wood waste incineration products [78]. F^−^ and Cl^−^ were the major components in the combustion products of plastic products. Acp, AcPy and Ant accounted for a significant proportion of the waste incineration products [79,80]. All of these supported Factor 4 as waste incineration source. Factor 5 had the highest loadings of SO_4_^2−^, NO_3_^−^ and NH_4_^+^ which were generated from the secondary conversion of SO_2_, NO_x_ and NH_3_. Ca^2+^ was also of large loading in Factor 5 and is the major component in construction dust. Therefore, Factor 5 represented the mixed source of secondary conversion and construction dust. In this study, these five sources were identified as the sources of major pollutants in Nanjing Jiangbei New Area from 2015 to 2019.

To further determine the relative contribution of the major sources to cytotoxic effects, multiple linear regressions were performed with standardized five factors and standardized cell viability, ROS levels and DNA damage levels, respectively. The results were displayed in Figure 6b. Except for the waste incineration source, the remaining four sources all contributed significantly to the cytotoxic effects (*p* < 0.05). In terms of cell viability, the mixed source of secondary conversion and construction dust contributed the most with 30.55%, followed by fossil fuel combustion source with 23.10%, traffic source with 22.27%, biomass combustion source with 14.71% and waste incineration source with 9.37%. For cellular ROS levels, the fossil fuel combustion source was the greatest contributor with 32.54%. Following that, the mixed source of secondary conversion and construction dust contributed 23.23%, traffic source contributed 16.32%, biomass combustion source contributed 14.73% and waste incineration source contributed 13.18%. The ranking of the contributions of the five sources to DNA damage levels was consistent with that to ROS levels. Fossil fuel combustion source contributed the most, followed by mixed source of secondary conversion and construction dust, traffic source, biomass combustion source and waste incineration source, with contributions of 35.12%, 24.88%, 16.06%, 15.07% and 8.87%, respectively. Overall, fossil fuel combustion, secondary conversion of pollutants and construction dust were the dominant sources of cytotoxic effects, contributing more than 50%. The traffic source and biomass combustion source also contributed a large proportion, which were worthy of attention in environmental management. They were the major sources of pollutants in Nanjing Jiangbei New Area from 2015 to 2019. Although many high-tech industries had been introduced, traditional chemical industries still existed and emitted large amounts of pollutants into the atmosphere, posing a potential threat to the health of surrounding residents. During the construction phase of the new area, dust from construction sites and exhaust from transport vehicles were almost daily present, so there might be a high risk of occupational exposure to construction workers, traffic police, drivers, etc. [81]. Straw burning had long been banned, but this phenomenon persisted in the suburbs. In addition, crop residues were often used as kitchen firewood in rural areas, which contributed to the emissions from biomass burning source [82].

## 4. Conclusions

In this study, PM_2.5_ was collected from October to November in 2015–2019 in Nanjing Jiangbei New Area, and the chemical fractions and toxicity to GC-2spd(ts) cells were determined. From 2015 to 2019, the mass concentration of PM_2.5_ showed an overall downward trend. Water-soluble ions were the most abundant component of PM_2.5_, and SNA accounted for the largest proportion of water-soluble ions, indicating the existence of severe secondary pollution at the sampling site. In this study, heavy metals were mainly from fossil fuel combustion and traffic emissions, WSOC from fossil fuel combustion and secondary conversion, and PAHs from combustion emissions from surrounding chemical parks. In terms of cytotoxicity, there was a dose–effect relationship between PM_2.5_ exposure and viability of GC-2spd(ts) cells. The strongest inhibition of cell viability was observed in 2016 and 2018, and the cytotoxicity of PM_2.5_ was relatively low in 2017 and 2019. The overall downward trends in ROS levels and DNA damage levels during these five years were probably due to similar trends in the concentrations of heavy metals and PAHs. This suggested that heavy metals and PAHs in PM_2.5_ were likely to be significant components causing oxidative stress and DNA damage in cells. The trends of ROS levels and DNA damage levels were not exactly consistent with those of cell mortality, implying that there were other mechanisms leading to cell death besides oxidative stress and DNA damage, which deserved further investigation in future studies. The results of this study are based on in vitro exposure experiments. The effects of PM_2.5_ on GC-2spd(ts) cells could reflect the reproductive toxicity risk of PM_2.5_ to some extent but could not completely restore the toxic effects of PM_2.5_ on the organism. Therefore, corresponding in vivo exposure studies are necessary for follow-up. By studying the changes in the reproductive system and germ cells after PM_2.5_ exposure in mice, the reproductive toxicity and toxicogenic mechanisms of PM_2.5_ can be further investigated. Five characteristic sources were extracted from pollutants at the sampling site for these five years, identified as fossil fuel combustion source, traffic source, biomass combustion source, waste incineration source and mixed source of secondary conversion and construction dust. Their contributions to the toxic effects of GC-2spd(ts) cells were evaluated. Fossil fuel combustion, secondary transformation of pollutants and construction dust were identified as the main contributors to cytotoxic effects. However, from the perspective of regional development, the contribution of an individual pollution source cannot be fixed, so this assessment approach has certain limitations. In addition, elevated levels of cellular ROS and DNA damage often lead to reduced cell viability, which may interfere with the assessment of source contribution. Therefore, studies on PM_2.5_ toxicity and source contribution assessment at more time points may be needed. Toxicity studies for different sources of PM_2.5_ could also be further conducted to compare their contributions. The reduction in PM_2.5_ concentration, cytotoxicity and toxic component concentrations in Nanjing Jiangbei New Area from 2015 to 2019 revealed that environmental improvement might benefit from industrial upgrading, and environmental policy played a considerable role as well.

## Figures and Tables

**Table 1:** Diagnostic ratios of PAHs in PM_2.5_ samples from 2015 to 2019.

Diagnostic Ratio	2015	2016	2017	2018	2019	Value	Sources
IND/(IND + B[ghi]P)						<0.20	Petroleum
	0.45	0.47	0.47	0.48	0.49	0.20–0.50	Diesel combustion
						>0.50	Coal combustion
B[a]A/(B[a]A + CHR)						<0.20	Petroleum
		0.20				0.20–0.35	Coal combustion
	0.37		0.35	0.35	0.38	>0.35	Vehicle exhaust
FL/(FL + Pyr)						<0.40	Petroleum
			0.45	0.44	0.44	0.40–0.50	Fuel combustion
	0.53	0.51				>0.50	Coal combustion
Ant/(Ant + PA)						<0.10	Petroleum
	0.31	0.48	0.46	0.20	0.37	>0.10	Combustion

## Data Availability

The data presented in this study are available on request from the corresponding author.

## Supplementary Materials

The following supporting information can be downloaded at: https://www.mdpi.com/article/10.3390/toxics11020092/s1. Figure S1. Location of the sampling site. Figure S2. Pearson correlation between cell viability, ROS levels and DNA damage levels of GC-2spd(ts) cells, and individual components of PM_2.5_. More detailed data is listed in the file named ‘Supplementary data.xlsx’. Figure S3 Mass fraction of major chemical compositions in PM_2.5_. Table S1. Instrumental conditions for microwave digestion and ICP-MS. Table S2. Instrumental conditions for ion chromatography. Table S3. Instrumental conditions for GC-MS. Table S4. The EF of metal elements in PM_2.5_ in Nanjing Jiangbei New Area from 2015 to 2019. Table S5. The total variance explained. Table S6. The rotated factor loading matrix ^a^.
