# Supplementary material for: Chemical Characteristics and Cytotoxicity to GC-2spd(ts) Cells of PM2.5 in Nanjing Jiangbei New Area from 2015 to 2019
Source: Toxics. 2023 Jan 18;11(2):92. doi: 10.3390/toxics11020092 (PMC9966943; doi:10.3390/toxics11020092)
Supplement: Supplementary file 1 [file toxics-11-00092-s001.zip › Supplementary data.pdf]

Chemical characteristics and cytotoxicity to GC-2spd(ts) cells of $PM_{2.5}$ in Nanjing Jiangbei New Area from 2015 to 2019

Pengxiang Ge [1], Zhengjiang Liu [2], Mindong Chen [1,*], Yan Cui [1], Maoyu Cao [1] and Xiaoming Liu [1]

[1] Jiangsu Key Laboratory of Atmospheric Environment Monitoring and Pollution Control, Collaborative Innovation Center of Atmospheric Environment and Equipment Technology, School of Environmental Science and Engineering, Nanjing University of Information Science & Technology, Nanjing 210044, China.

[2] Gansu Water Resources and Hydropower Survey and Design Research Institute, Lanzhou 730000, China.

* Correspondence: chenmd@nuist.edu.cn; Tel. +86-25-58731089.

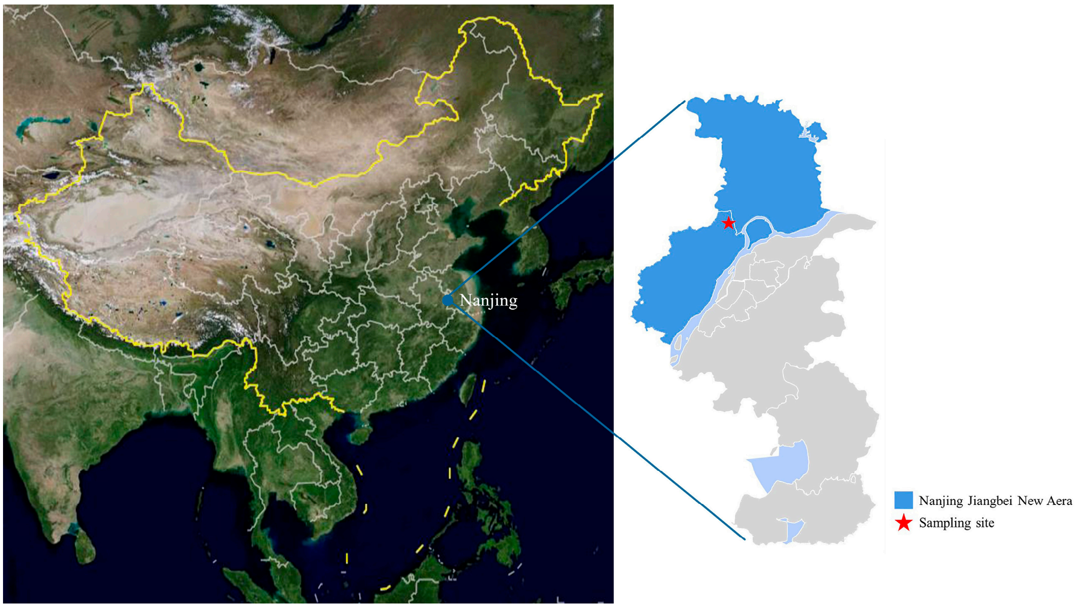

Figure S1. Location of the sampling site.

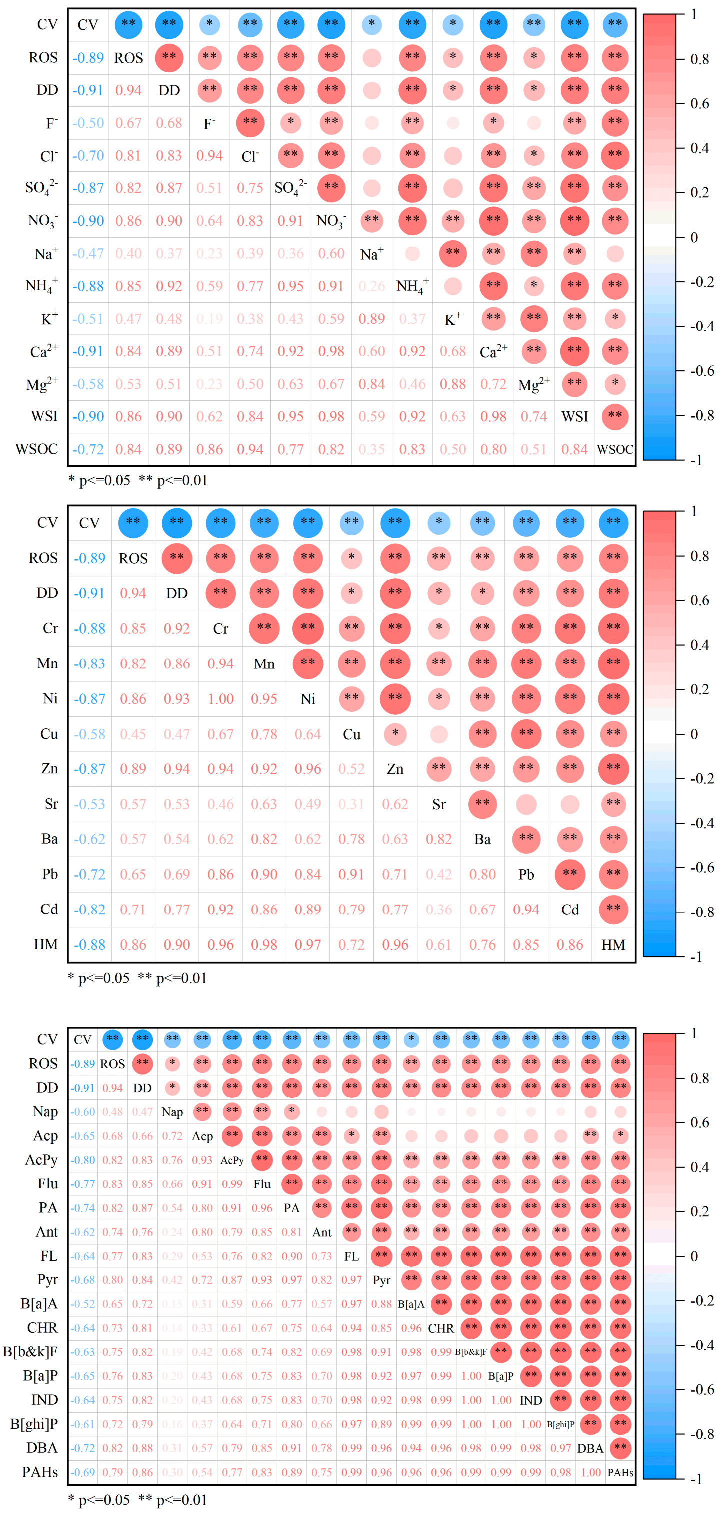

Figure S2. Pearson correlation between cell viability, ROS levels and DNA damage levels of GC-2spd(ts) cells, and individual components of $PM_{2.5}$. More detailed data is listed in the file named " Supplementary data.xlsx".

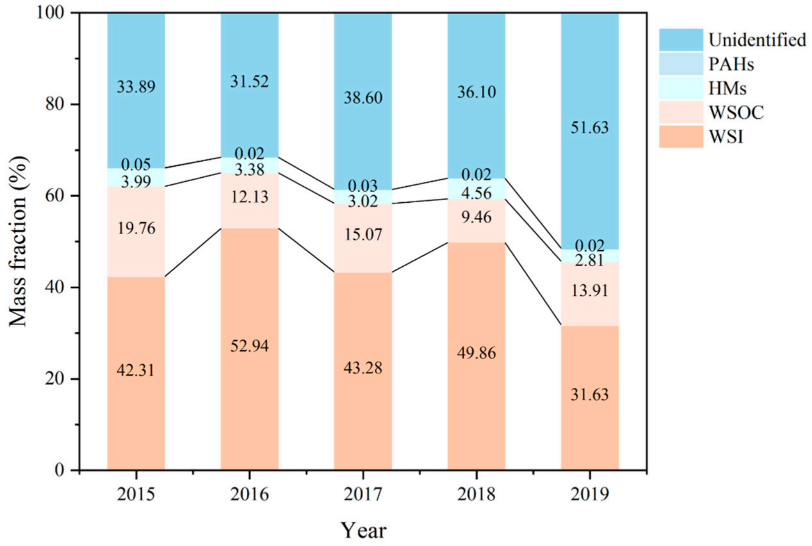

Figure S3 Mass fraction of major chemical compositions in $PM_{2.5}$.

Table S1. Instrumental conditions for microwave digestion and ICP-MS

	Parameters
Microwave dissolver	Mars-6, CEM, USA
Digestion solution	5mL HNO_3 (65%, GR)
Digestion temperature	170 ℃
Digestion time	30 min
ICP-MS RF power	1550W
Carrier gas	Ar
Cool gas flow rate	15 L/min
Aux gas flow rate	1 L/min
Nebulizer gas flow rate	1 L/min

Table S2. Instrumental conditions for ion chromatography

	Anion	Cation
Separation columns	Ion Pac (4×250 mm)	Ion Pac CS16 (4×250 mm)
Guard columns	Ion Pac AS11-HC (4×50 mm)	Ion Pac CG16 (4×50 mm)
Suppressors	ASRS-4 mm	CSRS-4 mm
Flow phases	10 mmol/L KOH	32 mmol/L methanesulfonic acid
Flow rates	1 mL/min	1 mL/min

Table S3. Instrumental conditions for GC-MS

	Parameters
Gas chromatography	Agilent 7890B
Mass spectrometer	Agilent 5977B
Solvent system	Dichloromethane
Volume of extraction solution	2 μL
Capillary column	Agilent HP-5 ms (30 m*0.25 mm*0.25 μm)
Column chamber temperature (the first 3min)	50℃
Column chamber heating rate	30 ℃/min
Column chamber temperature (the final)	325℃

Table S4. The EF of metal elements in $PM_{2.5}$ in Nanjing Jiangbei New Area from 2015 to 2019.

	background	2015	2016	2017	2018	2019
Mn	600.00	1.00	1.00	1.00	1.00	1.00
Ba	469.00	0.19	0.23	0.58	0.47	0.14
Sr	132.00	0.51	1.24	2.53	0.78	0.55
Cr	80.30	2.72	3.22	1.57	2.06	1.99
Ni	35.50	4.45	4.86	2.80	3.11	3.45
Pb	46.30	9.30	5.60	5.44	11.50	3.48
Cu	46.80	28.01	20.12	19.25	82.73	34.55
Zn	134.00	77.30	114.60	76.18	54.44	87.13
Cd	0.29	78.86	93.14	29.56	83.06	14.42

The calculation method is performed as follows:

$$EF = \frac{C_i/C_n}{X_i/X_n}$$

In the formula, C_i is the mass concentration of the target element in the atmosphere, C_n is the mass concentration of the selected reference element in the atmosphere, X_i is the mass concentration of the target element in the crust, and X_n is the mass concentration of the selected reference element in the crust. If the EF value is between 1 and 10, the element is considered to be contributed by a combination of anthropogenic and natural sources. If the EF is greater than 10, the element is considered to be mainly from anthropogenic sources. If the EF is less than 1, the element is considered to be mainly from natural sources. In this study, Mn was selected as the reference element. The soil background values for metal elements were obtained from China national environmental monitoring center.

Table S5. The total variance explained

Element	Initial eigenvalue			Square sum of extracted loadings			Square sum of rotated loadings		
	Total	% of Variance	Cumulative % of Variance	Total	% of Variance	Cumulative % of Variance	Total	% of Variance	Cumulative % of Variance
1	23.786	69.958	69.958	23.786	69.958	69.958	14.833	43.626	43.626
2	5.488	16.14	86.099	5.488	16.14	86.099	6.313	18.569	62.195
3	2.305	6.779	92.878	2.305	6.779	92.878	5.322	15.652	77.847
4	1.29	3.794	96.672	1.29	3.794	96.672	3.985	11.721	89.568
5	1.132	3.328	100	1.132	3.328	100	3.547	10.432	100
6	3.63E-15	1.07E-14	100						
7	2.16E-15	6.37E-15	100						
8	1.46E-15	4.29E-15	100						
9	1.01E-15	2.98E-15	100						
10	9.65E-16	2.84E-15	100						
11	9.06E-16	2.66E-15	100						
12	8.20E-16	2.41E-15	100						
13	7.20E-16	2.12E-15	100						
14	6.34E-16	1.87E-15	100						
15	5.48E-16	1.61E-15	100						
16	4.52E-16	1.33E-15	100						
17	3.39E-16	9.97E-16	100						
18	2.52E-16	7.42E-16	100						
19	1.14E-16	3.36E-16	100						
20	9.09E-17	2.67E-16	100						
21	-2.84E-17	-8.35E-17	100						
22	-9.83E-17	-2.89E-16	100						
23	-1.11E-16	-3.27E-16	100						
24	-2.33E-16	-6.86E-16	100						
25	-3.54E-16	-1.04E-15	100						
26	-3.63E-16	-1.07E-15	100						
27	-4.27E-16	-1.26E-15	100						
28	-5.79E-16	-1.70E-15	100						
29	-6.17E-16	-1.81E-15	100						
30	-6.77E-16	-1.99E-15	100						
31	-7.81E-16	-2.30E-15	100						
32	-8.29E-16	-2.44E-15	100						
33	-1.08E-15	-3.16E-15	100						
34	-2.82E-15	-8.30E-15	100						

Extraction method: principal component analysis.

Table S6. The rotated factor loading matrix [a]

Element	Factor1	Factor2	Factor3	Factor4	Factor5
F^-	0.836	0.148	0.083	0.514	-0.092
Cl^-	0.742	0.181	0.248	0.562	0.198
SO_4^{2-}	0.442	0.216	0.253	0.303	0.776

NO_3^-	0.498	0.521	0.342	0.319	0.512
Na^+	-0.073	0.403	0.886	0.208	-0.07
NH_4^+	0.597	0.35	-0.01	0.168	0.702
K^+	0.053	0.335	0.933	0.051	0.113
Ca^{2+}	0.451	0.458	0.453	0.165	0.596
Mg^{2+}	-0.053	0.182	0.81	0.411	0.373
WSOC	0.829	0.096	0.404	0.278	0.252
Cr	0.662	0.524	0.277	0.058	0.455
Mn	0.572	0.603	0.42	0.267	0.248
Ni	0.711	0.481	0.298	0.091	0.407
Cu	0.072	0.925	0.366	0.025	0.071
Zn	0.656	0.294	0.436	0.246	0.483
Sr	0.229	0.19	0.23	0.905	0.198
Ba	0.155	0.698	0.275	0.636	0.09
Pb	0.429	0.869	0.165	0.103	0.153
Cd	0.452	0.767	0.056	0.011	0.452
Nap	0.007	0.875	0.329	0.335	0.116
Acp	0.281	0.323	0.627	0.632	0.153
AcPy	0.523	0.476	0.433	0.504	0.244
Flu	0.621	0.376	0.479	0.45	0.201
PA	0.755	0.318	0.509	0.23	0.132
Ant	0.612	-0.157	0.465	0.536	0.312
FL	0.951	0.204	0.144	0.144	0.112
Pyr	0.867	0.237	0.329	0.282	0.072
B[a]A	0.98	0.169	-0.082	0.042	0.044
CHR	0.935	0.118	-0.088	0.057	0.318
B[b&k]F	0.959	0.139	-0.011	0.12	0.216
B[a]P	0.958	0.14	0.031	0.09	0.23
IND	0.96	0.144	0.011	0.111	0.212
B[ghi]P	0.966	0.138	-0.045	0.085	0.196
DBA	0.919	0.179	0.168	0.174	0.254

Extraction method: principal component analysis.

Rotation Method: Varimax with Kaiser Normalization.

[a] The rotation converged after 7 iterations.
